# Unresolved Systemic Inflammation, Long COVID, and the Common Pathomechanisms of Somatic and Psychiatric Comorbidity

**DOI:** 10.3390/jcm11175114

**Published:** 2022-08-30

**Authors:** Chih-Sung Liang, Piotr Gałecki, Kuan-Pin Su

**Affiliations:** 1 Department of Psychiatry, Beitou Branch, Tri-Service General Hospital, National Defense Medical Center, Taipei 112, Taiwan; 2 Department of Psychiatry, National Defense Medical Center, Taipei 114, Taiwan; 3 Department of Adult Psychiatry, Medical University of Lodz, 91-229 Lodz, Poland; 4 College of Medicine, China Medical University, Taichung 404, Taiwan; 5 Mind–Body Interface Laboratory (MBI-Lab), China Medical University and Hospital, Taichung 404, Taiwan; 6 Depression Center and Department of Psychiatry, An-Nan Hospital, China Medical University, Tainan 709, Taiwan

## 1. Introduction

Monoamine hypothesis is an overestimated hypothesis of major depressive disorder (MDD), and the treatments and biomarkers that target it only show modest effect in randomized controlled trials+ (RCTs) or case–control studies [1]. Accumulating evidence has implicated inflammation dysregulation in the pathophysiology of MDD, highlighting complex interactions among the mind, brain, and body [2]. The inflammation theory may explain a high occurrence of somatic symptoms and physical illness in certain subtypes of MDD.

The coronavirus disease 2019 (COVID-19) pandemic may have exacerbated the burden of MDD. A global survey study reports that the COVID-19 pandemic provoked 53.2 million new cases of MDD (a 27.6% increase) [3]. One-fifth of patients recovering from the acute phase have persistent somatic and neuropsychiatric symptoms and signs related to the long-term effects of COVID-19 infection (also known as long COVID or post-acute COVID-19 syndrome) [4,5].

Research suggests that COVID-19 may induce various neuropsychiatric manifestations via massive inflammatory processes [6,7]. In other words, the neuropsychiatric manifestations of long COVID might be the largest clinical observation of inflammation theory for depression in the coming years.

## 2. Common Pathomechanisms of Somatic and Psychiatric Comorbidity

The current Special Issue in the *Journal of Clinical Medicine* is dedicated to collecting high-quality studies that explore the common pathophysiology of somatic and psychiatric comorbidities for MDD, and its association with the inflammation theory of MDD. Inflammatory cytokines have an impact on monoamine neurotransmission, which is important to mood regulation [8]. Cytokines can break down tryptophan into kynurenine, and nicotinamide adenine dinucleotide (NAD) is an important coenzyme in the kynurenine pathway [8]. A study reported that genetic variation in NAD homeostasis was associated with the occurrence of MDD. Cytokines orchestrate hematopoiesis via Janus kinase (JAK) and signal transducer and activator of transcription (STAT) pathway, regulating inflammation and the immune process [9]. A study compared depressed patients and healthy controls in the mRNA and protein expression of genes for pathways of JAK1-JAK3 and STAT1-STAT5 [10], and found increased expression of JAK3 and decreased expression of STAT1 in the depressed patients. In addition to inflammation, imbalance in oxidative stress has been implicated in the pathophysiology of MDD [11,12]. Paraoxonases and myeloperoxidase are two proteins involved in the balance of oxidative stress [13]. A study examined the expression of these two biomarkers at the mRNA and protein levels in depressed patients and controls [14].

For patients with MDD, environmental triggers (e.g., stress, unhealthy diet, poor sleep, and physical inactivity) activate peripheral inflammatory responses, such as induction of cytokine release and activation of different immune cells. The increased levels of peripheral cytokines can be transmitted to the brain by traversing the BBB, vagus nerve, or gut microbiota–brain signaling. The central–peripheral immune crosstalk further influences the neurocircuitries, monoamines, oxidative stress, and hypothalamic–pituitary–adrenal (HPA) axis in patients with MDD [8,15,16]. These pathophysiological processes may elucidate the treatment response, cognitive impairment, and somatic and psychiatric comorbidities for MDD [8,15,16,17].

Stressful event exposure is usually followed by psychological resilience and then recovery. The trajectory and treatment response of these new-onset cases of depression requires direct assessment methodology. In clinical practice, around half of patients experiencing their first episode of MDD may not achieve full remission during first-line treatment [18]. Treatment-resistant depression (TRD) is most often defined as an inadequate response to at least two treatments with adequate dosages and durations. The prevalence of TRD in the pandemic context is unknown. Culture and context may affect the prevalence, symptomatology, and longitudinal trajectories of MDD and, as expected, long COVID depression [18,19]. Moreover, evidence on new treatments (e.g., esketamine nasal spray, repetitive transcranial magnetic stimulation, psilocybin) may not be available in every country [20]. Studies are still needed to assess the benefits of the cognitive behavioral approach [21] or complementary therapy (e.g., music, mindfulness) [22] for patients with co-occurring physical and psychological symptoms, such as inflammation or long COVID.

## 3. Long COVID, Neuroinflammation, and Somatic and Psychiatric Comorbidity

**Figure 1:**
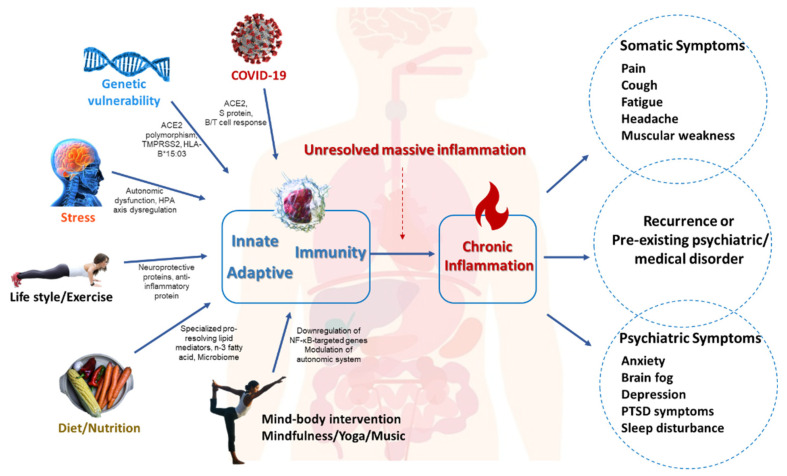
Potential mechanisms linking inflammation theory of MDD to long COVID. ACE2: Angiotensin-converting enzyme 2; HLA-B: Human Leukocyte Antigen-B; HPA: Hypothalamic-pituitary-adrenal; NF-KB: Nuclear factor kappa B.

Figure 1 illustrates the potential mechanisms linking the inflammation theory of MDD with long COVID. We believe that understanding the shared theory of neuroinflammation between the two may help future research into mind–body intervention for the protean manifestations of long COVID. Depression is a common neuropsychiatric symptom of long COVID, and around 90% of patients may develop at least one neuropsychiatric symptom 6 months following COVID-19 infection [5]. Several mechanisms have been proposed to explain neuropsychiatric sequelae long COVID, including static brain injury, immune–inflammation aberrations, viral persistence in tissue reservoirs, or reactivation of other latent viruses [4,5]. Importantly, the immune–inflammation aberrations have been implicated in the pathophysiology of MDD [8,15,16]. A postmortem study found patients with moderate or severe COVID-19 infection had increased levels of interleukin (IL)-6, which is known to cause blood–brain barrier (BBB) disruption [23]. Wachowska et al. showed increased serum levels of IL1-β and IL-6 and impaired episodic memory in depressed patients compared with controls [24]. The same group also reported increased serum levels of IL-1 and IL-6 in the depressed group compared with the control group [25].

Cognitive impairment (known as brain fog) is also a common symptom of long COVID. A meta-analysis reported that 22% of patients may develop cognitive impairment 12 weeks after the COVID-19 infection [26]. The suggested mechanisms of cognitive impairment after COVID-19 infection were direct viral encephalitis, neuroinflammation, BBB damage, hypoxia, and cerebrovascular disease [26,27]. Neuroinflammation has been suggested as a mechanism linking the etiology of MDD with its neurocognitive symptoms [17,28]. A recent study reported that higher coping flexibility was associated with a lower risk of depression [29]. Another study found that coping flexibility mediated the association between personality and psychological stress [30]. However, the association between depression and cognitive impairment in long COVID remains understudied. Future research can investigate whether patients with higher cognitive ability can be immune to depression after COVID-19 infection, or whether patients with severe COVID-19 infection may be more vulnerable to cognitive impairment and depression than those with mild or asymptomatic COVID-19 infection.

COVID-19 infection can have prolonged effects on multiple organs and the brain [4,26,27]. Long COVID consists of a variety of somatic symptoms, such as fatigue, muscular weakness, cough, chest pain, headache, and brain fog. Available data showed that the prevalence of depression 12 weeks after COVID-19 infection was 11–20% in the hospitalized population and 3–16% in the nonhospitalized population [5]. In fact, MDD can also present with somatic symptoms (e.g., fatigue) and can co-occur with a wide range of physical disorders (e.g., obesity, diabetes, cardiovascular disease, and chronic pain) [31]. A recent study suggested that the risk of depression was associated with a noninvasive test used to predict liver fibrosis [32]. Another study suggested that serum levels of lipid correlated with depressive symptoms in men depending on the levels of cholesterol [33]. To date, few studies have examined the interrelationship between depression and somatic symptoms in the context of long COVID.

## 4. Conclusions

Unresolved massive inflammation may be a shared mechanism between MDD and somatic and psychiatric comorbidity. Targeting inflammation is a potential therapeutic strategy for depression, which consists of a variety of mild and persistent somatic and neuropsychiatric symptoms. The long-term impact of COVID-19 infection on the brain and extrapulmonary organs also emphasizes the brain–body connection. Rigor and reproducibility of scientific research are required to elucidate the somatic and neuropsychiatric sequelae of long COVID. The current Special Issue provided several new pieces of the etiological, diagnostic, and therapeutic puzzle of MDD. We believe these findings will inspire future research on the role of inflammation in the pathomechanisms of depression.
